# Regional Analysis of Intact and Defective HIV Proviruses in the Brain of Viremic and Virally Suppressed People with HIV

**DOI:** 10.1002/ana.26750

**Published:** 2023-08-12

**Authors:** Thomas A. Angelovich, Catherine R. Cochrane, Jingling Zhou, Carolin Tumpach, Sarah J. Byrnes, Janna Jamal Eddine, Emily Waring, Kathleen Busman-Sahay, Claire Deleage, Trisha A. Jenkins, Anna C. Hearps, Stuart Turville, Paul R. Gorry, Sharon R. Lewin, Bruce J. Brew, Jacob D. Estes, Michael Roche, Melissa J. Churchill

**Affiliations:** 1 Infectious and Inflammatory Diseases, School of Health and Biomedical Sciences, RMIT University, Melbourne, Victoria, Australia; 2 Life Sciences Discipline, Burnet Institute, Melbourne, Victoria, Australia; 3 Department of Infectious Diseases, The University of Melbourne at the Peter Doherty Institute for Infection and Immunity, Melbourne, Victoria, Australia; 4 Department of Medicine, The Royal Melbourne Hospital, The University of Melbourne, Melbourne, Victoria, Australia; 5 Vaccine and Gene Therapy Institute, Oregon Health & Science University, Hillsboro, Oregon, USA; 6 AIDS and Cancer Virus Program, Leidos Biomedical Research Inc., Frederick National Laboratory for Cancer Research, Frederick, Maryland, USA; 7 Central Clinical School, Monash University, Melbourne, Victoria, Australia; 8 The Kirby Institute, University of New South Wales, Kensington, New South Wales, Australia; 9 Department of Infectious Diseases, Alfred Hospital and Monash University, Melbourne, Victoria, Australia; 10 Victorian Infectious Diseases Service, Royal Melbourne Hospital at the Peter Doherty Institute for Infection and Immunity, Melbourne, Victoria, Australia; 11 Peter Duncan Neurosciences Unit, Departments of Neurology and Immunology St. Vincent’s Hospital, University of New South Wales, Sydney, New South Wales, Australia; 12 University of Notre Dame, Sydney, New South Wales, Australia; 13 Departments of Microbiology and Medicine, Monash University, Melbourne, Victoria, Australia

## Abstract

Here, we provide the first regional analysis of intact and defective HIV reservoirs within the brain. Brain tissue from both viremic and virally suppressed people with HIV (PWH) harbored HIV *pol* DNA in all regions tested, with lower levels present in basal ganglia and cerebellum relative to frontal white matter. Intact proviruses were primarily found in the frontal white matter but also detected in other brain regions of PWH, demonstrating frontal white matter as a major brain reservoir of intact, potentially replication competent HIV DNA that persists despite antiretroviral therapy.

HIV persistence in cellular and tissue reservoirs is the major barrier to HIV cure. During acute infection, HIV enters the brain via infiltrating T cells and monocytes, leading to viral dissemination in microglia, perivascular macrophages, astrocytes, and pericytes.^1,2^ We and others have also shown that viral DNA persists in the brain of virally suppressed people with HIV (PWH) on antiretroviral therapy (ART) at similar levels to those present during untreated infection.^3,4^ Recently, we utilized the intact proviral DNA assay (IPDA) to further characterize the brain reservoir, providing the first evidence of intact and defective HIV proviruses in the frontal white matter of PWH.^3^ Importantly, levels of intact proviruses did not appear to be impacted by long-term viral suppression, supporting the presence of a stable and potentially replication competent HIV reservoir in the brain. This study focused on HIV persistence in the frontal white matter due to the key role that this region plays in the pathogenesis of HIV-associated neurocognitive disorders (HAND),^5^ which affects ~40% of virally suppressed PWH.^6^ Studies using non-human primate models of HIV and earlier analyses of autopsy brain tissue from PWH with HIV-associated dementia have also reported regional differences in the size and activity of the HIV reservoir in the brain.^7,8^ Differences in the intact and defective HIV reservoir throughout regions of the brain may reflect more significant roles in potential central nervous system (CNS) escape or domain-specific cognitive impairment. Therefore, understanding the proviral landscape across the brain, particularly the presence of intact proviruses in virally suppressed PWH, is essential for defining the effect of HIV persistence on brain health in PWH.

## Methods

### Tissue Cohort

Matched fresh frozen frontal white matter, basal ganglia, and cerebellum brain tissue from PWH or HIV-seronegative individuals was provided by the National NeuroAIDS Tissue Consortium (NNTC, USA; https://nntc.org^9^) and processed with ethics approval (RMIT University, Australia; HREC#20843).

### Quantification of HIV Reservoirs in Brain Regions

The gDNA was extracted from brain tissue using the AllPrep DNA/RNA/miRNA universal kit (QIAGEN, Hilden, Germany) for HIV *pol* quantification or the DNA extraction kit (Agilent, Santa Clara, CA) for the IPDA, as previously described.^3^ HIV *pol* and ribonuclease P/MRP Subunit P30 (*RPP30*) for total input gDNA quantification was quantified, as previously described.^10^ A median (interquartile range [IQR]) of 502,725 (IQR = 405,526 – 686,992) cells were screened for HIV *pol* DNA analysis and 510,292 (IQR = 401,101 – 610,438) screened by IPDA per tissue region per patient. Intact (Ψ^+^ and *env*^+^) and defective (Ψ^+^ or *env*^+^) HIV genomes were measured by the IPDA, as previously described.^3^ Median droplet shearing index (DSI) was 0.24 (IQR = 0.19–0.30), with DSI levels similar between frontal white matter tissue and other sites.

### Statistics

Comparisons were made using nonparametric Mann–Whitney *U* (unpaired) or Wilcoxon tests (paired) using GraphPad Prism (version 9.2.0; GraphPad Software, La Jolla, CA).

## Results

### HIV DNA Is Present in Basal Ganglia and Cerebellum at Lower Levels than Frontal White Matter Tissue in Virally Suppressed PWH

**FIGURE 1:**
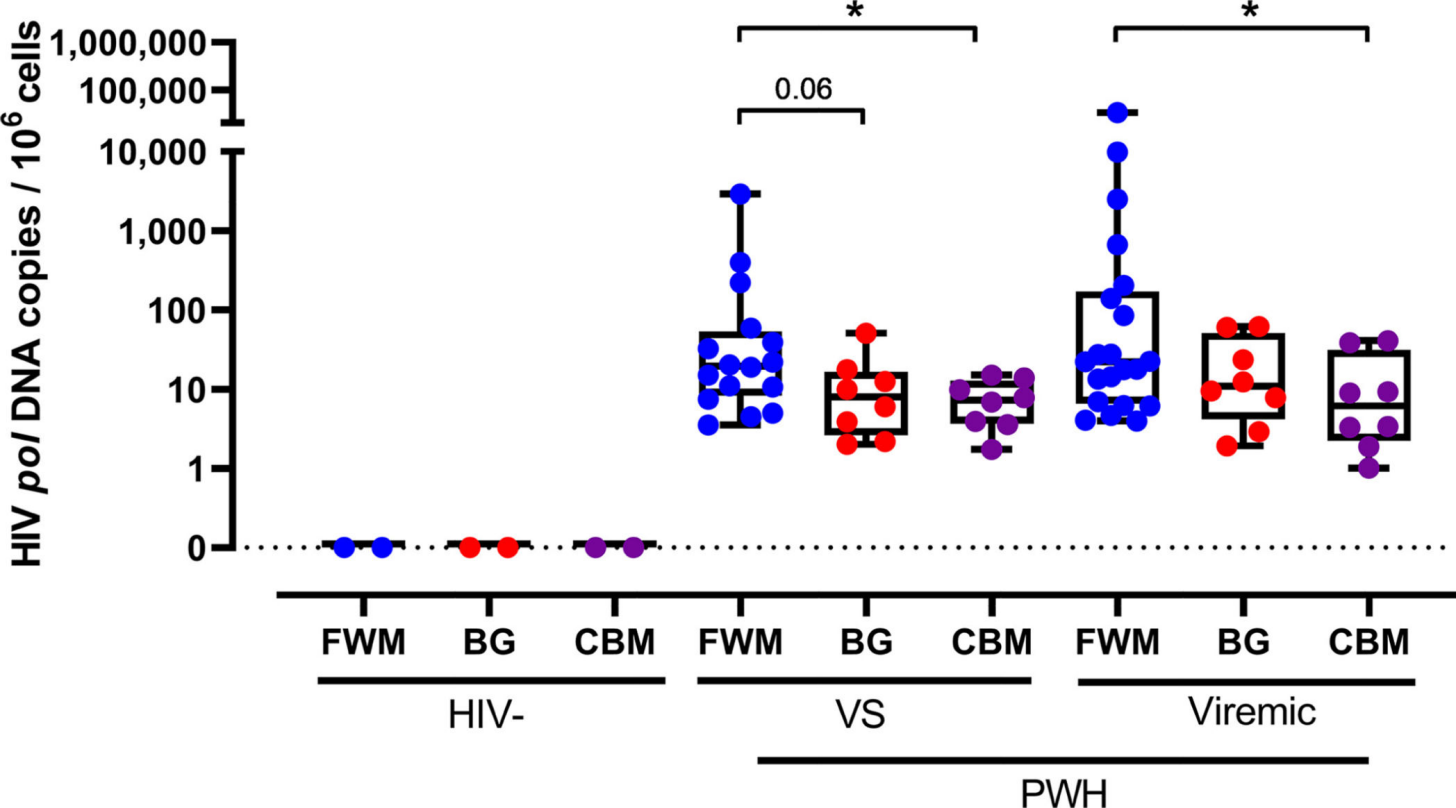
Quantification of HIV *pol* DNA in human brain regions. HIV *pol* DNA was quantified in fresh frozen FWM (*blue*), BG (*red*), and CBM ( *purple*) tissue from VS (n = 16) and untreated PWH (viremic; n = 21) or HIV-seronegative controls (HIV-; n = 2) by ddPCR. HIV *pol* DNA copies standardized to 10^6^ cell equivalents. Median and IQRs shown. Comparisons made using non-parametric Mann–Whitney *U* tests. *p < 0.05. BG = basal ganglia; CBM = cerebellum; ddPCR = droplet digital polymerase chain reaction; FWM = frontal white matter; IQR = interquartile range; PWH = people with HIV; VS = virally suppressed.

To characterize HIV persistence throughout the brain, fresh frozen frontal white matter, cerebellum, and basal ganglia tissue from virally suppressed or untreated PWH were assessed for HIV *pol* DNA by ddPCR (Supplementary Table S1). Viral suppression was defined as > 12 months of continuous ART treatment with undetectable viral load (one blip < 250 copies/ml was allowed > 6 months prior to sampling unless stated in Supplementary Table S1). HIV *pol* DNA was detected in all brain regions from all subjects (Fig 1), indicating widespread HIV persistence. HIV *pol* DNA was detected in the cerebellum at a lower level than in frontal white matter tissue in both viremic and virally suppressed PWH (both *p* < 0.05). Basal ganglia from virally suppressed PWH also showed a trend to lower levels of HIV *pol* DNA than in the frontal white matter ( *p* = 0.06). HIV DNA was not detected in HIV-seronegative controls. Levels of HIV *pol* DNA in each region were similar between virally suppressed and viremic PWH, indicating that long-term viral suppression did not affect the distribution of the HIV reservoir.

### Frontal White Matter Is a Major Reservoir of Intact Proviral DNA Relative to Other Brain Regions in Virally Suppressed PWH

**FIGURE 2:**
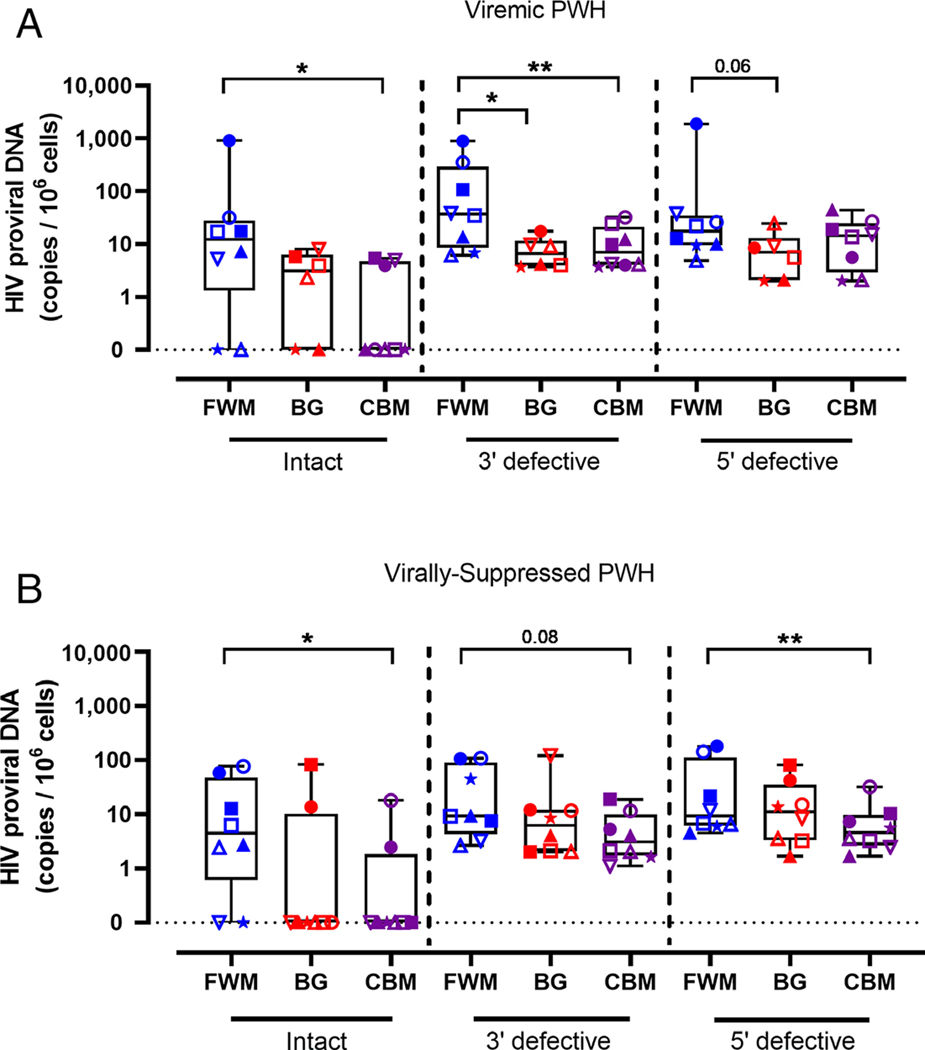
Intact and defective proviral landscape across multiple brain regions in PWH. (A) Intact (Ψ^+^*env*^+^), 3′ defective (Ψ^+^*env*^−^), and 5′ defective (Ψ^−^*env*^+^) HIV proviruses were quantified in matched frozen human FWM (*blue*; n = 8), BG (*red*; n = 6), or CBM ( *purple*; n = 8) from viremic PWH by IPDA. Proviral copy number was standardized to 10^6^ cells. (B) Proviral copy number of intact, 3′ defective, and 5′ defective proviral HIV DNA in FWM (n = 8), BG (n = 8), and CBM (n = 8) from virally suppressed PWH. Symbols represent individuals. Comparisons made using nonparametric paired Wilcoxon tests. *p < 0.05, **p < 0.01. BG = basal ganglia; CBM = cerebellum; FWM = frontal white matter; IPDA = intact proviral DNA assay; PWH = people with HIV; VS = virally suppressed.

We next used the IPDA to characterize intact and defective HIV DNA across the frontal white matter, basal ganglia, and cerebellum from matched PWH. The IPDA is a multiplex droplet digital polymerase chain reaction (ddPCR) approach that utilizes fluorescent primer/probe sets targeting either HIV *psi* (Ψ) or HIV envelope (*env*) regions at alternate ends of the genome.^3,11^ Therefore, detection of proviral DNA expressing both fluorophores is considered intact, and potentially capable of viral replication. The IPDA was performed on viremic PWH (n = 8), virally suppressed PWH (n = 8) or HIV-seronegative controls (n = 2) where matched tissue was available. Two basal ganglia tissues were excluded from IPDA analysis due to suboptimum levels of DSI (> 0.50; Supplementary Table S1). Intact HIV proviral genomes were present in the frontal white matter in 6 of 8, cerebellum in 3 of 8 and basal ganglia tissue in 4 of 6 viremic PWH (Fig 2A). Frontal white matter tissue harbored higher levels of intact HIV DNA relative to the cerebellum (*p* < 0.05). Frontal white matter also had higher levels of 3′ defective HIV DNA than other brain regions in viremic PWH (both *p* < 0.05 relative to frontal white matter) and a trend to higher levels of 5′ defective proviral DNA in basal ganglia was observed. As expected, the majority of proviral DNA in each region were defective (either 3′ or 5′ defective proviruses; > 80% of total proviruses for all regions). No HIV DNA was detected in HIV-seronegative controls (n = 2; data not shown).

Similar to findings in viremic PWH, the majority of virally suppressed PWH tested contained intact proviral DNA in the frontal white matter (6 of 8; Fig 2B). However, contrary to findings in viremic PWH, comparatively few virally suppressed PWH had detectable intact proviral HIV DNA in cerebellum (2 of 8) or basal ganglia (2 of 8), demonstrating that the frontal white matter is a more stable reservoir of intact proviral DNA following long-term viral suppression. One PWH contained intact proviral DNA in each region (see solid circle in Fig 2B). Frontal white matter also contained higher levels of intact proviral DNA than other regions (median [IQR]: frontal vs basal ganglia vs cerebellum; 4.46 [0.61–47.0] vs. 0.00 [0.00–10.3] vs. 0.00 [0.00–1.85]; see Fig 2B), indicating that of the brain regions analyzed in this study the frontal white matter is the major HIV reservoir in the brain of virally suppressed PWH.

## Discussion

Our study provides the first characterization of the intact and defective proviral HIV reservoir across multiple regions of the brain of virally suppressed PWH. HIV *pol* DNA was detected in cerebellum, basal ganglia, and frontal white matter of virally suppressed PWH which supports widespread viral persistence throughout the brain in line with earlier studies in viremic and ART-treated PWH.^4,12–14^ Although these studies did not find a statistically significant difference in HIV *gag* DNA levels between basal ganglia and frontal white matter tissue, we find higher levels of HIV *pol* DNA in the frontal white matter relative to the other regions tested. These differences may relate to cohort differences including viremia status, length of ART-suppression, presence/absence of HIVE, types of ART drugs used, and use of HIV *pol* instead of *gag* as a measure of HIV DNA. Moreover, we used a stringent definition of viral suppression with individuals being suppressed for a median of 4.1 years prior to autopsy.

Importantly, frontal white matter tissue harbored the most intact HIV proviral DNA relative to the other 2 brain regions tested. Intact proviral DNA was also detected, albeit infrequently, in other regions with 2 of 8 virally suppressed PWH harboring intact proviral DNA in basal ganglia or cerebellum, respectively, with one virally suppressed PWH harboring intact proviral DNA in each region. The relatively limited number of PWH harboring intact proviral DNA in the basal ganglia and cerebellum may result from these sites containing a smaller and/or more labile HIV reservoir. Basal ganglia and cerebellum are also rich in gray matter made up of neuronal cell bodies (known to not be receptive to HIV infection) and are more permissive to ART penetration than frontal white matter.^15^ In contrast, there is a higher density of HIV permissive microglia in frontal white matter tissue than either basal ganglia or cerebellum,^16^ which may impact the capacity for HIV to infect and persist in gray matter at levels seen in the frontal white matter. It is possible that treatment regimen may impact regional distribution of intact proviral reservoirs in the brain, however, this would need to be assessed in larger studies powered to address this question. The IPDA cannot distinguish between integrated and unintegrated forms of HIV DNA, however, a recent study demonstrated that the majority of HIV DNA in the brain is integrated (n = 63).^14^ Whether unintegrated forms of HIV DNA are intact in the brain remains unclear.

The majority of the HIV reservoir in frontal white matter, basal ganglia, and cerebellum tissue contained defective proviruses which mimics findings from other cellular and tissue HIV reservoirs.^3,11,17,18^ Defective proviral DNA may be capable of producing viral proteins with potential neurotoxic effects, which may lead to ongoing immune activation and neuropathology throughout the brain.^19,20^

Finally, in line with our previous work and other studies in frontal white matter,^3,18^ no difference in the levels of HIV intact and defective proviral genomes were observed between viremic and virally suppressed PWH across all regions. Together these data support recent evidence of the persistence of an intact, potentially replication competent HIV reservoir in the brain that is not eradicated by ART.^21^ Therefore, future studies need to consider the impact of both intact and defective proviral DNA (and potentially RNA) throughout the brain of virally suppressed PWH on the underlying pathogenesis of HAND in PWH.

## Supplementary Material

Supplement Table 1
