# Supplementary material for: Regional Analysis of Intact and Defective HIV Proviruses in the Brain of Viremic and Virally Suppressed People with HIV
Source: Ann Neurol. Author manuscript; Available in PMC 2024 Mar 5. (PMC10914117; doi:10.1002/ana.26750)
Supplement: Supplement Table 1 [file NIHMS1957518-supplement-Supplement_Table_1.docx]

| **Supplementary Table 1.**  **Clinical cohort characteristics** | | | | | | | | | | | | |
| --- | --- | --- | --- | --- | --- | --- | --- | --- | --- | --- | --- | --- |
| **Donor** | **Age** | **Sex** | **Yrs VS** | **Plasma VL** | **CD4 count** | **Nadir CD4 mm^3^** | **HIV *pol* DNA** | | | **IPDA** | | |
|  |  |  |  |  |  |  | **FWM** | **BG** | **CBM** | **FWM** | **BG** | **CBM** |
| **Viremic PWH** | |  |  |  |  |  |  |  |  |  |  |  |
| *nVS HIV+ 1* | 39 | M | - | 570 | 79 | 1 | Y^b^ | Y | Y | Y^b^ | Y | Y |
| *nVS HIV+ 2* | 61 | M | - | 275 | 340 | 17 | Y | Y | Y | Y | Y | Y |
| *nVS HIV+ 3* | 52 | F | - | <50 | 471 | 190 | Y | Y | Y | Y | - ^c^ | Y |
| *nVS HIV+ 4* | 54 | F | - | <50 | 8 | ^d^ | Y^b^ | Y | Y | Y^b^ | Y | Y |
| *nVS HIV+ 5* | 44 | F | - | 14286 | 290 | ^d^ | Y^b^ | Y | Y | Y^b^ | Y | Y |
| *nVS HIV+ 6* | 49 | M | - | 750000 | 3 | ^d^ | Y^b^ | Y | Y | Y^b^ | Y | Y |
| *nVS HIV+ 7* | 42 | M | - | 688 | 441 | 105 | Y^b^ | Y | Y | Y^b^ | Y | Y |
| *nVS HIV+ 8* | 43 | M | - | 64 | 110 | 31 | Y | Y | Y | Y | - ^c^ | Y |
| *nVS HIV+ 9* | 56 | M | - | 61223 | 24 | 8 | Y |  |  |  |  |  |
| *nVS HIV+ 10* | 60 | M | - | <50 | 38 | 38 | Y |  |  |  |  |  |
| *nVS HIV+ 11* | 64 | F | - | 359 | 72 | 11 | Y^b^ |  |  |  |  |  |
| *nVS HIV+ 12* | 57 | M | - | 40133 | 299 | ^d^ | Y^b^ |  |  |  |  |  |
| *nVS HIV+ 13* | 40 | F | - | 157009 | 5 | ^d^ | Y^b^ |  |  |  |  |  |
| *nVS HIV+ 14* | 42 | M | - | 25022 | 17 | 4 | Y^b^ |  |  |  |  |  |
| *nVS HIV+ 15* | 46 | M | - | >750000 | 6 | 6 | Y^b^ |  |  |  |  |  |
| *nVS HIV+ 16* | 37 | M | - | 72125 | 1 | 0 | Y^b^ |  |  |  |  |  |
| *nVS HIV+ 17* | 40 | M | - | >750000 | 14 | ^d^ | Y |  |  |  |  |  |
| *nVS HIV+ 18* | 41 | M | - | 500000 | 37 | ^d^ | Y |  |  |  |  |  |
| *nVS HIV+ 19* | 46 | M | - | 17500 | ^d^ | ^d^ | Y |  |  |  |  |  |
| *nVS HIV+ 20* | 35 | M | - | 2827 | 211 | ^d^ | Y |  |  |  |  |  |
| *nVS HIV+ 21* | 52 | M | - | 1618 | 171 | ^d^ | Y |  |  |  |  |  |
| ***Median (IQR)*** | **45 (40-55)** |  |  | **1618 (140 – 40133)** | **72 (11 – 267)** | **19 (8.75 – 76.5)** |  |  |  |  |  |  |
| **Virally suppressed PWH** | |  |  |  |  |  |  |  |  |  |  |  |
| *VS HIV+ 1^a^* | 57 | M | 6.44 | UD | 48 |  | Y | Y | Y | Y | Y | Y |
| *VS HIV+ 2* | 39 | M | 1.46 | UD | 112 | 17 | Y | Y | Y | Y | Y | Y |
| *VS HIV+ 3* | 59 | F | 2.47 | UD | 328 | 161 | Y^b^ | Y | Y | Y^b^ | Y | Y |
| *VS HIV+ 4* | 58 | M | 2.57 | UD | 213 | ^d^ | Y^b^ | Y | Y | Y^b^ | Y | Y |
| *VS HIV+ 5* | 64 | M | 3.4 | UD | 1043 | 60 | Y^b^ | Y | Y | Y^b^ | Y | Y |
| *VS HIV+ 6* | 46 | M | 3.76 | UD | 61 | 61 | Y | Y | Y | Y | Y | Y |
| *VS HIV+ 7* | 60 | M | 2.15 | UD | 497 | 28 | Y | Y | Y | Y | Y | Y |
| *VS HIV+ 8* | 67 | M | 7.32 | UD | 355 | 30 | Y^b^ | Y | Y | Y^b^ | Y | Y |
| *VS HIV+ 9* | 52 | M | 6.15 | UD | 417 | 78 | Y^b^ |  |  |  |  |  |
| *VS HIV+ 10* | 66 | M | 5.02 | UD | 465 | ^d^ | Y^b^ |  |  |  |  |  |
| *VS HIV+ 11* | 62 | M | 11.6 | UD | 274 | 65 | Y |  |  |  |  |  |
| *VS HIV+ 12* | 44 | M | 4.5 | UD | 785 | 206 | Y |  |  |  |  |  |
| *VS HIV+ 13* | 64 | M | 2.54 | UD | 798 | 318 | Y |  |  |  |  |  |
| *VS HIV+ 14* | 52 | M | 2.58 | UD | 383 | 230 | Y |  |  |  |  |  |
| *VS HIV+ 15* | 63 | M | 9.15 | UD | 140 | 54 | Y |  |  |  |  |  |
| *VS HIV+ 16* | 56 | M | 6.21 | UD | 239 | 90 | Y^b^ |  |  |  |  |  |
| ***Median (IQR)*** | **59 (52 – 64)** |  | **4.1 (2.5 – 6.4)** | **UD** | **341 (158 – 489)** | **65 (42 – 184)** |  |  |  |  |  |  |
| *BG: basal ganglia; CBM: cerebellum; FWM: frontal white matter; IPDA: intact proviral DNA assay; nVS: non-virally suppressed; PWH: people with HIV; UD; undetectable; VS: virally suppressed*  *^a^ Patient had one blip at 656 HIV RNA copies/mL > 4 months from death; ^b^ Previously reported in (3); ^c^ Excluded from IPDA analyses (droplet shearing index >0.50); ^d^ missing data* | | | | | | | | | | | | |
